# Structural and Functional Analysis of Nonheme Iron Enzymes BCMO-1 and BCMO-2 from *Caenorhabditis elegans*

**DOI:** 10.3389/fmolb.2022.844453

**Published:** 2022-02-10

**Authors:** Weimin Pan, Yong-Ling Zhou, Jian Wang, Huai-En Dai, Xiao Wang, Lin Liu

**Affiliations:** ^1^ School of Life Sciences, Anhui University, Hefei, China; ^2^ Anhui Key Laboratory of Modern Biomanufacturing, Anhui University, Hefei, China

**Keywords:** carotenoid, dioxygenase, β-propeller, retinoid, crystallography

## Abstract

Carotenoid metabolism is critical for diverse physiological processes. The nematode *Caenorhabditis elegans* has two genes that are annotated as β-carotene 15,15′-monooxygenase (BCMO) and are 17 centimorgan apart on chromosome II, but the function of BCMO-1 and BCMO-2 remains uncharacterized. Sequence homology indicates that the two enzymes belong to the carotenoid cleavage dioxygenase family that share a seven-bladed β-propeller fold with a nonheme iron center. Here we determined crystal structures of BCMO-1 and BCMO-2 at resolutions of 1.8 and 1.9 Å, respectively. Structural analysis reveals that BCMO-1 and BCMO-2 are strikingly similar to each other. We also characterized their β-carotene cleavage activity, but the results suggest that they may not act as β-carotene 15,15′-oxygenases.

## 1 Introduction

Carotenoids are tetraterpene pigments naturally synthesized by plants and certain microorganisms, and are essential nutrients for animals (Walter and Strack, 2011; Harrison and Quadro, 2018). Their metabolic products participate in a variety of physiological processes such as retinoid signaling and vision cycle, and exert health-beneficial effects with antioxidant properties (von Lintig, 2010; Álvarez et al., 2014). Cleavage of carotenoids are catalyzed by carotenoid cleavage dioxygenases (CCDs) that share a seven-bladed β-propeller topology with a nonheme iron coordinated by four absolutely conserved histidines (Harrison and Bugg, 2014). The iron activates the dioxygen by a single electron transfer and generates the substrate radical intermediate, which then returns one electron to iron and forms the carbocation intermediate that ultimately yields aldehyde products (Sui et al., 2013; Guengerich and Yoshimoto, 2018).

The CCD family members are distributed from archaea to mammals and have diverse functions beyond carotenoid cleavage (Auldridge et al., 2006; Lobo et al., 2012). β-Carotene 15,15′-oxygenase (BCO1) (von Lintig and Vogt, 2000; Redmond et al., 2001) and β-carotene 9′,10′-oxygenase (BCO2) (Kiefer et al., 2001) are the two typical CCDs. Retinal pigment epithelium-specific 65 kDa protein (RPE65), a CCD member, acts as a *cis*-*trans* retinoid isomerase of the polyene chain (Jin et al., 2005). Structural studies have been carried out on apocarotenoid-15,15′-oxygenase (ACO) from the cyanobacterium *Synechocystis* sp. PCC 6803 (Kloer et al., 2005), RPE65 from the ox *Bos taurus* (Kiser et al., 2009), 9-*cis*-epoxycarotenoid dioxygenase viviparous-14 (VP14) from the maize *Zea mays* (Messing et al., 2010), and CCD from the soil archaeon *Nitrosotalea devanaterra* (NdCCD) (Daruwalla et al., 2020). The diverse functions of CCDs are attributed to the unique structural variations in the helical dome that covers the active center in the β-propeller domain (Daruwalla and Kiser, 2020). However, no structure of BCO1 or BCO2 from any animals has been reported until now, while their kinetics and substrate specificity have been investigated (Lindqvist and Andersson, 2002; dela Seña et al., 2013; Kelly et al., 2018; Bandara et al., 2021).

**FIGURE 1:**
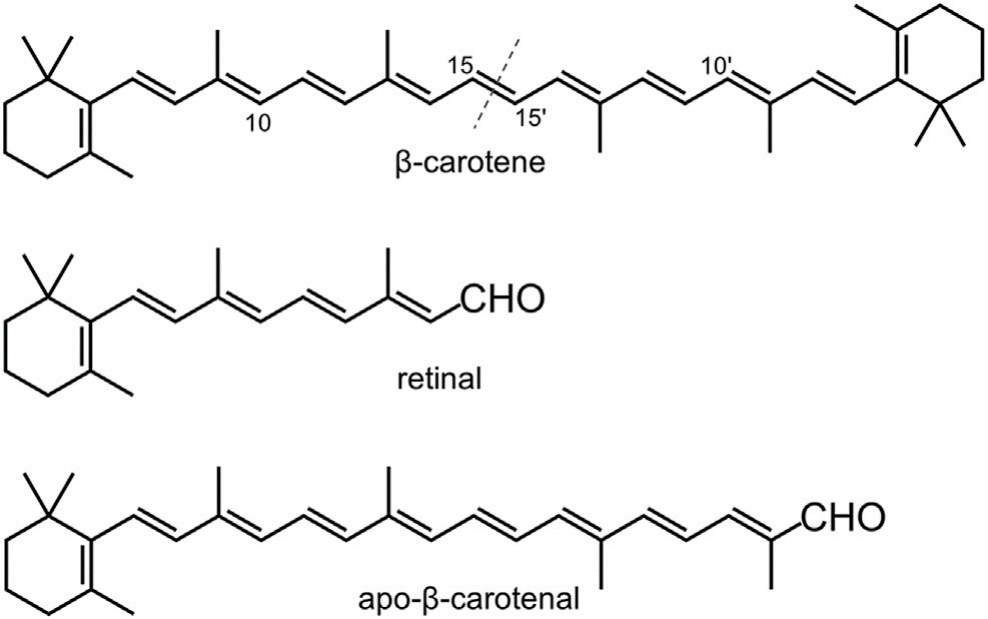
Chemical structures of β-carotene, retinal, and apo-β-carotenal. The dashed line indicates the symmetric cleavage site. Apo-β-carotenal is used for enzymatic activity test.

The genome of nematode *Caenorhabditis elegans* has two CCD genes, which are 17 centimorgan apart on chromosome II and are annotated as *bcmo-1* and *bcmo-2* in WormBase. BCMO stands for β-carotene 15,15′-monooxygenase, although human BCO1 is actually a dioxygenase (dela Seña et al., 2014). As the name implies, the BCMO proteins are predicted to cleave β-carotene symmetrically (Figure 1). Here we use the annotated name in line with previous studies on *C. elegans*, in which BCMO-1 has been found to participate in cadmium-induced retinoic acid signaling (Cui et al., 2007; Cui and Freedman 2009). This work describes the crystal structures of BCMO-1 and BCMO-2. Compared with ACO, RPE65, VP14, and NdCCD, these two nematode proteins are most similar to the isomerase RPE65. *In vitro* activity assay did not detect their proposed ability for β-carotene cleavage, suggesting that they play other role than acting as BCO per se.

## 2 Materials and Methods

### 2.1 Protein Expression and Purification

The *C. elegans bcmo-1* and *bcmo-2* genes (Y46G5A.24 and F53C3.12 in WormBase) were codon-optimized for expression in *Escherichia coli*, synthesized, and delivered in pUC57 by Sangon Biotech (Shanghai, China). Each gene was amplified by PCR and inserted between the restriction sites NdeI and XhoI of pET28a(+) (Novagen). The resulting two plasmids encode the 529-residue BCMO-1 and 530-residue BCMO-2, respectively, which are preceded by an N-terminal His6 tag. Each plasmid was transformed into *E. coli* BL21(DE3) cells (Novagen). The cells were grown at 37°C in LB medium containing 30 μg/ml kanamycin, and when the optical density at 600 nm reached 0.6∼0.8, expression was induced by 0.4 mM isopropyl β-D-thiogalactopyranoside. The cells were grown at 16°C for additional 18 h before harvested by centrifugation. The cell pellets were resuspended in lysis buffer (0.3 M NaCl and 20 mM Tris-Cl, pH 7.5) plus 20 mM imidazole and lyzed by sonication at 0°C. The lysate was cleared by centrifugation. The supernatant was loaded onto a nickel-chelating column (QIAGEN) and washed with the lysis buffer plus 20 mM imidazole. The protein was eluted with 200 mM imidazole in lysis buffer. The sample was concentrated by ultrafiltration for further purification by size exclusion chromatography (SEC) with a HiLoad 16/60 Superdex 200 column (GE Healthcare), which was equilibrated and eluted with the lysis buffer. The purity of the fractions was analyzed by SDS–PAGE. Expression and purification of mouse BCO1 was performed under the same condition.

### 2.2 Crystallization

Crystal trays were set up at 16°C using the sitting-drop vapor diffusion method. Drops were prepared by mixing 1 μl of purified protein and 1 μl of reservoir solution against 150 μl reservoir solution. The purified BCMO-1 was concentrated to 20 mg/ml in lysis buffer. Crystals were obtained using the reservoir solution of 0.2 M ammonium sulfate, 0.1 M Bis-Tris, pH 5.5, and 25% (w/v) polyethylene glycol 3,350. The purified BCMO-2 was concentrated to 36 mg/ml in a buffer containing 0.2 M NaCl and 20 mM Tris-Cl, pH 7.5, and crystals were obtained using the reservoir solution of 0.2 M ammonium acetate and 2.2 M ammonium sulfate. Before synchrotron data collection, the crystals were soaked in reservoir solution with 15% (v/v) glycerol and flash-cooled in liquid nitrogen.

### 2.3 Structure Determination and Structure Analysis

X-ray diffraction data (Table 1) were recorded on beamlines BL18U1 and BL19U1 of the National Facility for Protein Sciences in Shanghai (Zhang et al., 2019). Data were processed using the HKL-3000 program package (Minor et al., 2006). The phase of BCMO-1 structure was solved by molecular replacement using Phaser (McCoy et al., 2007) in the CCP4 suite (Winn et al., 2011), with the coordinates of *B. taurus* RPE65 (PDB entry 4RSE) being used as search model (Kiser et al., 2015). Structural refinement was performed using Coot (Emsley and Cowtan, 2004) and PHENIX (Afonine et al., 2012). The structure of BCMO-2 was solved using the BCMO-1 structure as model. Model quality was evaluated by MolProbity (Chen et al., 2010). The atomic coordinates and structure factors of BCMO-1 and BCMO-2 have been deposited in the Protein Data Bank under the accession codes 7WH0 and 7WH1, respectively. Tunnel analysis was performed with the toolkit MOLE (Pravda et al., 2018) and the result was imported into PyMOL (Schrödinger, LLC) for visualization. Alignment was performed with Clustal Omega (Madeira et al., 2019) and the output file was drawn using ESPript (Gouet et al., 2003).

**TABLE 1:** Data collection and refinement statistics.

Protein	BCMO-1	BCMO-2
PDB code	7WH0	7WH1
Data collection		
Space group	P12_1_1	I4_1_22
Wavelength (Å)	0.9792	0.9792
Resolution (Å)	50–1.80 (1.86–1.80)	50–1.90 (1.97–1.90)
Unit cell		
a, b, c (Å)	73.2, 104.7, 76.4	148.8, 148.8, 136.3
α, β, γ (°)	90, 102.2, 90	90, 90, 90
Total reflections	685107 (64934)	1586788 (116653)
Unique reflections	102264 (9990)	58459 (4402)
Redundancy	6.7 (6.5)	26.4 (26.5)
Completeness (%)	98.2 (96.6)	100 (100)
I/σI	29.1 (3.0)	41.7 (2.3)
*R*_*merge*_ ^a^	0.066 (0.621)	0.093 (2.078)
*R*_*pim*_ ^b^	0.027 (0.259)	0.018 (0.409)
*CC*_*1/2*_	0.997 (0.887)	0.999 (0.733)
Wilson B-factor	28.25	19.83
Refinement		
Resolution (Å)	31.63–1.80 (1.86–1.80)	39.49–1.90 (1.97–1.90)
*R*_*work*_ ^c^ ^/^ *R*_*free*_ ^d^	0.166/0.189	0.158/0.186
No. of molecules	2	1
No. of atoms	9473	4638
Protein	8546	4029
Ligand	26	47
Water	901	508
Average B (Å^2^)	35.32	27.35
Protein	34.73	25.34
Ligand	34.50	49.52
Water	40.22	39.90
R.m.s deviations		
Bond lengths (Å)	0.007	0.007
Bond angles (°)	0.89	0.84
Ramachandran plot		
Favored (%)	96.58	96.54
Allowed (%)	3.42	3.46

Values in parentheses are for highest resolution shell.

a *R*_merge_ = ∑_hkl_∑_i_|*I*_i_(hkl) − < *I*(hkl) > |/∑_hkl_∑_i_ *I*_i_(hkl), where *I*_i_(hkl) is the *i*th observation of reflection hkl and <*I*(hkl)> is the weighted intensity for all observations *i* of reflection *hkl*.

b *R*_*pim*_ = *R*_*merg*e_[*1/*(*N-1*)]^1/2^.

c *R*_work_ = ∑| |*F*_o_| − |*F*c||/∑|*F*_o_|*,* where *F*_o_ and *F*_c_ are the observed and calculated structure factors, respectively.

d R_free_ is the cross-validated R factor computed for a test set of 5% of the reflections, which were omitted during refinement.

### 2.4 Enzymatic Activity Assay and HPLC Analysis

The activity assay was performed based on established methods (Sui et al., 2015). Two typical CCD substrates, β-carotene and apo-β-carotenal (Figure 1), were used to test the enzymatic activity of BCMO-1 and BCMO-2. The positive control was mouse BCO1, which can cleave β-carotene and apo-β-carotenal and yield retinal (Redmond et al., 2001; Poliakov et al., 2005; Amengual et al., 2013). The recombinant mouse BCO1 protein was obtained in a similar manner to that used for BCMO-1 and BCMO-2. The *trans*-β-carotene was obtained from Sigma-Aldrich; *trans*-β-apo-8′-carotenal and retinal were purchased from Shanghai Aladdin Biochemical Tech; no further purification was performed. Assays were run at a final volume of 200 μl consisting of 0.1 M Tris-HCl, pH 7.5, 0.5 mM dithiothreitol, 5% Tween 40, 4 mM sodium cholate, 15 mM nicotinamide, and 18-μg substrate (β-carotene or apo-β-carotenal), with 2.5 mM (final) enzyme being added last to initiate the reaction. Reactions were kept in the dark at 37°C for 12 h before being quenched by addition of 50 μl of 37% formaldehyde. Each quenched reaction was mixed with 250-μl solvent A (methanol/water, 90/10, v/v) and 250-μl solvent B (propan-2-ol/acetonitrile/water, 72/18/10, v/v/v), and was then filtered using a 0.22 μm membrane to remove insoluble substrates. HPLC was performed using a Hypersil ODS C-18 column (4.0 × 250 mm, Thermo Scientific) on a Shimadzu 20A system equipped with SPD-M40 UV-Vis detector. 40 μl out of ∼750-μl filtered sample was injected into the column at a flow rate of 1 ml/min and at a column temperature of 28°C. Separation was performed using the elution profile: 100% solvent A (0–5 min), 100% solvent B (5–30 min), and 100% solvent A (30–40 min). Separation was monitored at 380 and 448 nm.

## 3 Results

### 3.1 BCMO-1 Structure

**FIGURE 2:**
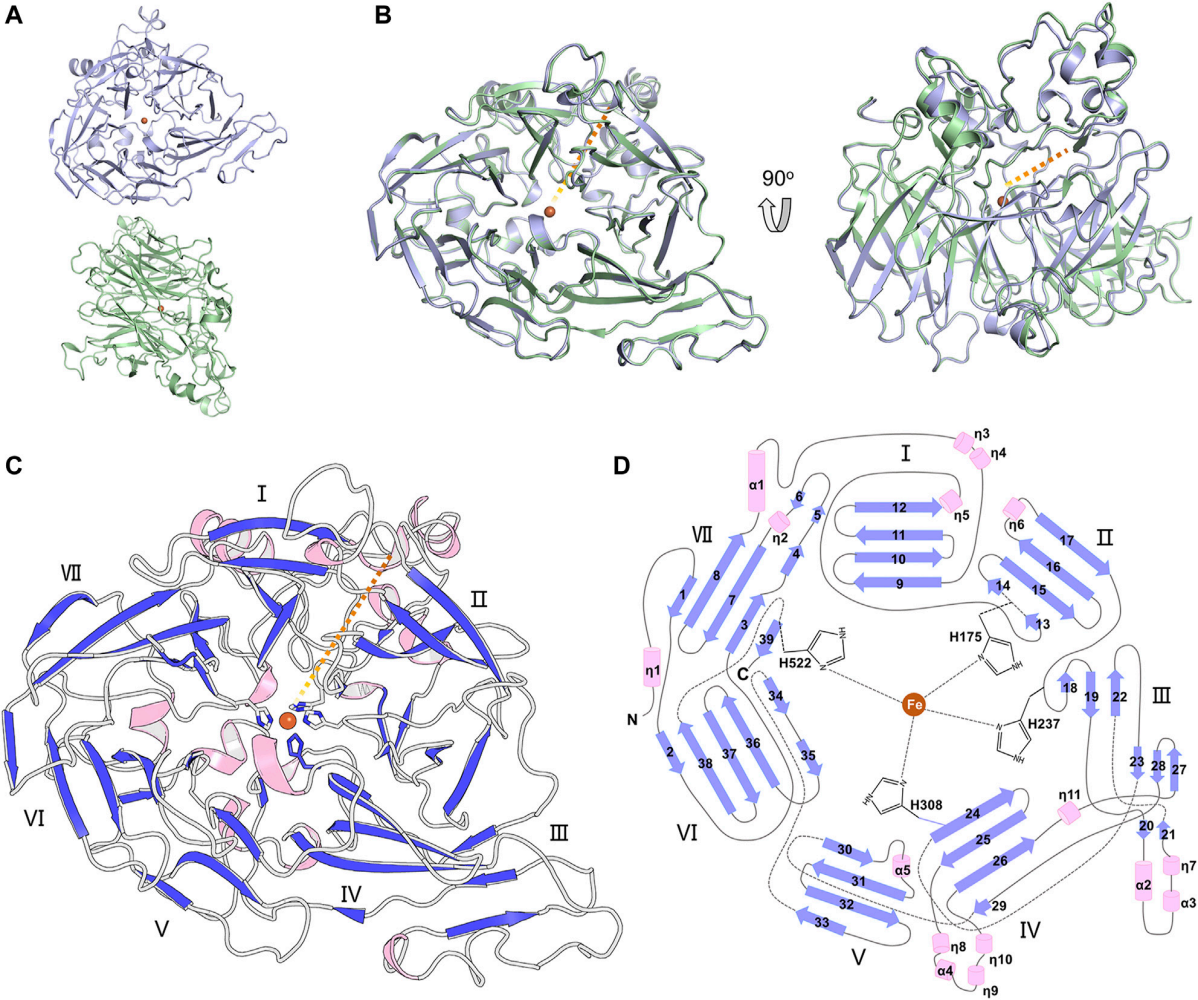
Overall structure of BCMO-1. **(A)** BCMO-1 dimer in crystal. Protein is in ribbon representation with chain A in light purple and chain B in light green. The iron ion is shown as sphere and colored in brown. **(B)** Superimposition of chains A and B. The orange dashed line indicates the tunnel to the active center. **(C)** Secondary structural elements of BCMO-1. α-Helices and η(3_10_)-helices are in light pink; β-strands are in purple blue. The seven β-propellers are labeled. The side chains of the iron-coordinating His are shown in sticks. **(D)** Topology diagram depicting the secondary structural elements and the iron-coordinating His.

The full-length BCMO-1 with an N-terminal His-tag was heterogenously expressed and purified by nickel affinity chromatography and then SEC. Purified BCMO-1 was crystallized and the diffraction data were collected to 1.8-Å resolution (Table 1). The structure was determined by molecular replacement, and two BCMO-1 molecules (chains A and B) were found in an asymmetric unit (Figure 2A). The two chains are highly similar with a root-mean-square deviation (RMSD) of 0.20 Å, with the large differences occurring in the helical dome (Figure 2B) and reflecting the relative flexibility of these regions. A substrate access tunnel is located at the interface among the dome, blades I and II. Chain A is used for tertiary structure analysis and secondary structure definition (Figure 2C). BCMO-1 has 39 β-stands, 5 α-helices and 11 3_10_-helices as defined by the dictionary of secondary structure of proteins (DSSP) algorithm (Kabsch and Sander, 1983). The iron is coordinated by His175, His237, His308, and His522 from blades II, III, IV, and VII, respectively (Figure 2D).

**FIGURE 3:**
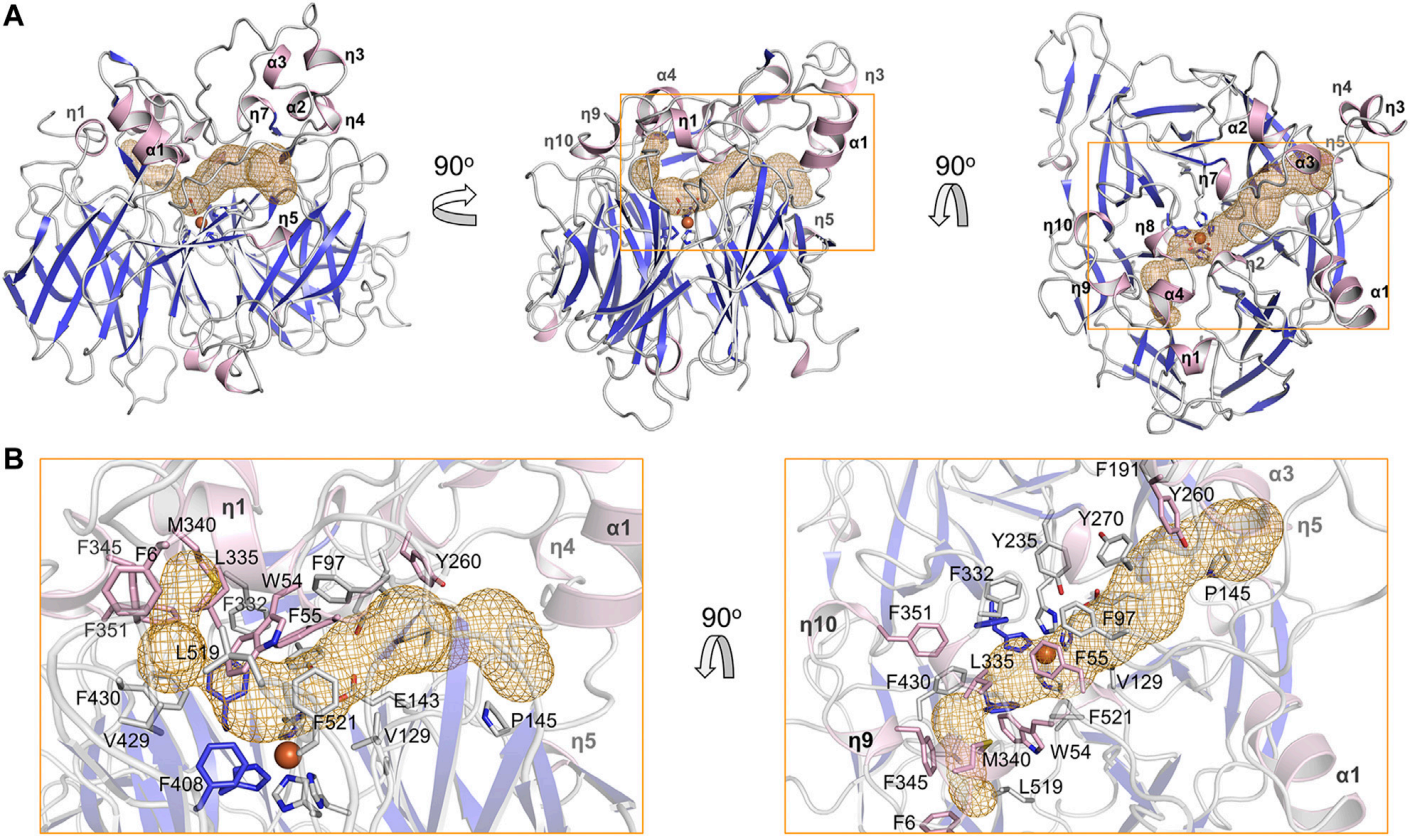
Substrate tunnel of BCMO-1. **(A)** Ribbon representation of BCMO-1 and mesh representation of the substrate tunnel. The color scheme is as in Figure 1C; the tunnel is in orange; boxes denote zoomed regions shown in B. The orientation in the left panel is same to that in Figure 1B (right panel). **(B)** Zoomed-in view of the tunnel. Ribbons are in transparence; side chains of Phe6, Trp54, Phe55, Phe97, Val129, Glu143, Pro145, Phe191, Tyr235, Tyr260, Tyr270, Phe307, Phe332, Leu335, Met340, Phe345, Phe351, Phe408, Val429, Phe430, Leu519, Phe521, and the iron-coordinating His are shown as sticks.

The substrate tunnel is a bent cavity passing below the helical dome (Figure 3A). A narrow neck unequally divides the tunnel into two parts. The main part of tunnel (also indicated by the orange dashed line in Figure 2) runs from the entrance located between helices η4 and η5 to the iron center; the minor part heads to the interface between helices η1 and α4, which is likely the exit passage of the product. Hydrophobicity of the tunnel is ensured by abundant lipophilic residues especially aromatic residues (Figure 3B). Trp54 and Phe430 clamp the neck of the tunnel. The distance from the neck to the entrance along the main part of tunnel is approximate 30 Å, which is long enough to accommodate a carotene molecule.

### 3.2 BCMO-2 Structure

**FIGURE 4:**
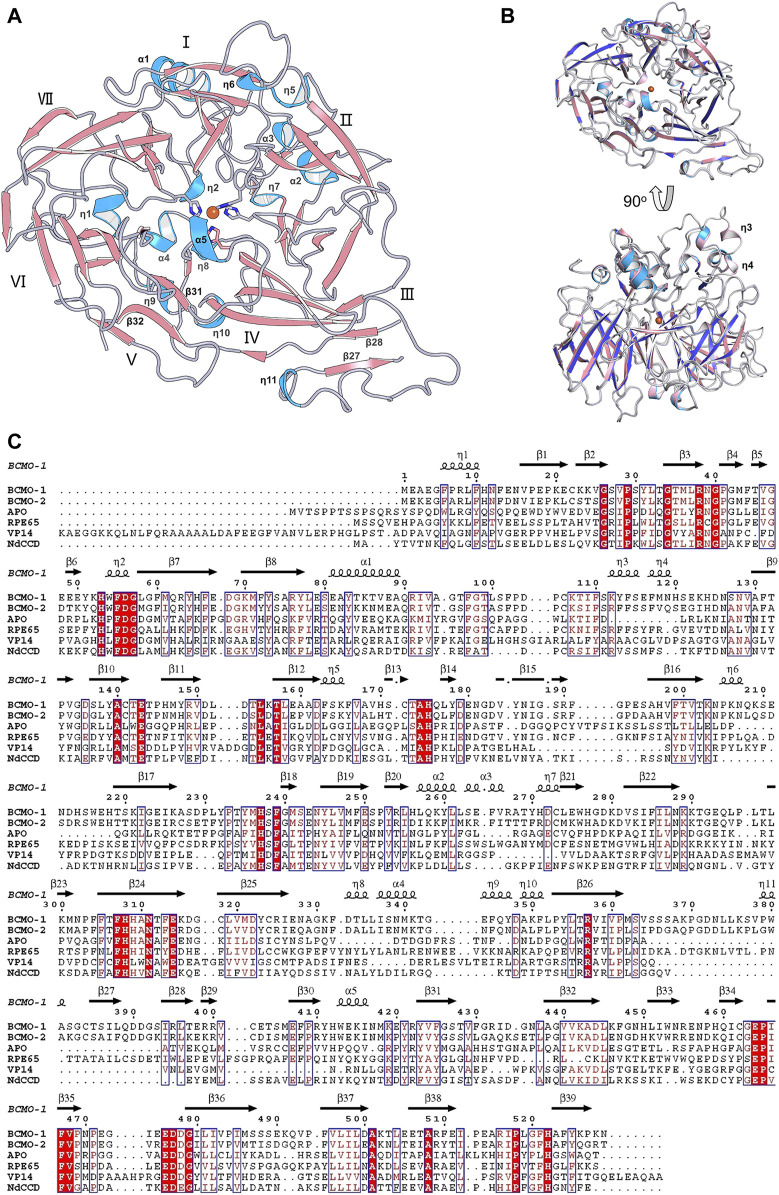
Overall structure of BCMO-2 and comparison with BCMO-1. **(A)** Secondary structural elements of BCMO-2. α-Helices and η-helices are in light blue and are labeled; β-strands are in light red. β-Strands 27, 28, 31, and 32 are labeled. **(B)** Superimposition of BCMO-1 and BCMO-2. BCMO-1 is colored as in Figure 2 η-Helices 3 and 4 that are not observed in BCMO-2 are labeled. **(C)** Amino acid sequence alignment of BCMO-1, BCMO-2, ACO, RPE65, VP14, and NdCCD. The alignment figure was created by ESPript. Conserved residues are in red background, and similar residues are in red and boxed. Secondary structural elements of BCMO-1 are shown on the top of the alignment.

The full-length BCMO-2 was produced in same way as for BCMO-1, and was crystallized and diffracted to 1.9 Å (Table 1). BCMO-2 has one molecule in an asymmetric unit and comprises 39 β-stands, 5 α-helices and 9 3_10_-helices (Figure 4A). Compared with BCMO-1, two 3_10_-helices (η3 and η4 in the loop connecting α1 and β9) are not observed due to missing density for residues Pro103–Gly122 (Figure 4B). Residues Asp390–Ser392 from the β27–β28 loop and residues Leu430–Glu436 from the β31–β32 loop are not observed either. The β31–β32 loop hosts Ser435, the only insertion in BCMO-2 (530 residues in total) relative to BCMO-1 (529 residues). The boundaries of secondary structural elements are identical between BCMO-2 and BCMO-1 except that the numbering after residue Ser435 differs by one residue (Figure 4C). The RMSD value between BCMO-2 and BCMO-1 (chain A) is 0.39 Å for 429 Cα atoms aligned, indicating that these two structures are highly similar. Therefore, consistent nomenclature of secondary structural elements is used for these two enzymes.

**FIGURE 5:**
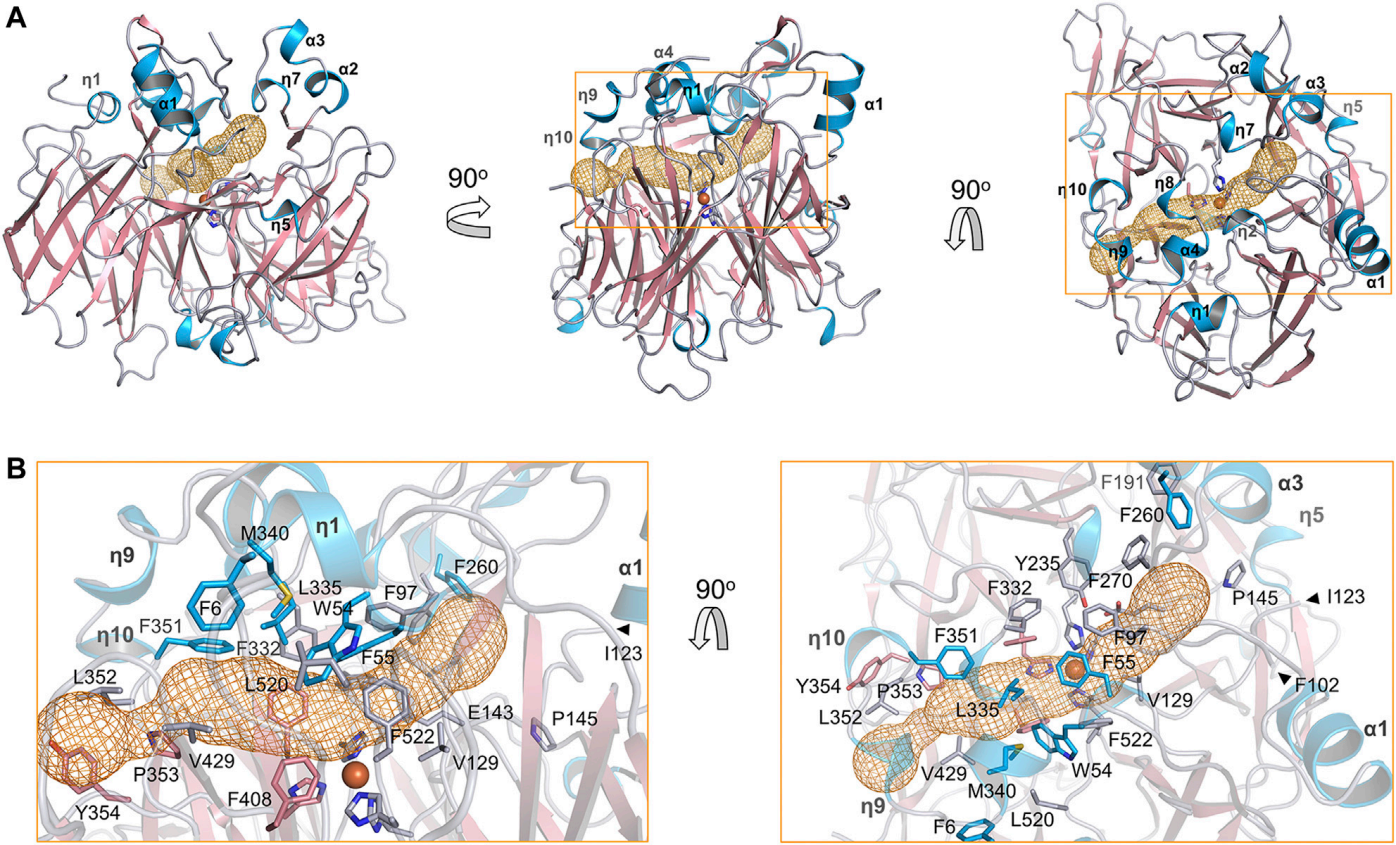
Substrate tunnel of BCMO-2. **(A)** Ribbon representation of BCMO-2 and mesh representation of the tunnel. The color scheme is as in Figure 3. Box denotes the zoomed region shown in B. **(B)** Zoomed-in view of the tunnel. Ribbons are in transparence; side chains of Phe6, Trp54, Phe55, Phe97, Val129, Glu143, Pro145, Phe191, Tyr235, Phe260, Phe270, Phe307, Phe332, Leu335, Met340, Phe351–Tyr354, Phe408, Val429, Leu520, Phe522, and the iron-coordinating His are shown as sticks. The triangles denote Phe102 and Ile123, the ends of traced backbone between domains VII and I.

The substrate tunnel of BCMO-2 passes through the helical dome (Figure 5A). Its two ends are flanked by two unobserved fragments, Pro103–Gly122 and Leu430–Glu436, respectively. Helices η3 and η4 observed in BCMO-1 are located within the first fragment, whose aromatic residues should provide a potential membrane-binding site adjacent to the tunnel. Because of missing electron density, the tunnel entrance that should be located between helices η4 and η5 shrinks inward (Figure 5B). The second missing fragment hosts Leu430, the counterpart of BCMO-1 Phe430, and its lack of electron density allows the tunnel runs towards η9 and η10. Despite differences at the two ends, the central part of the BCMO-2 tunnel is highly similar to that of BCMO-1, as revealed by the conserved hydrophobic residues.

### 3.3 BCMO-1 and BCMO-2 Active Centers

**FIGURE 6:**
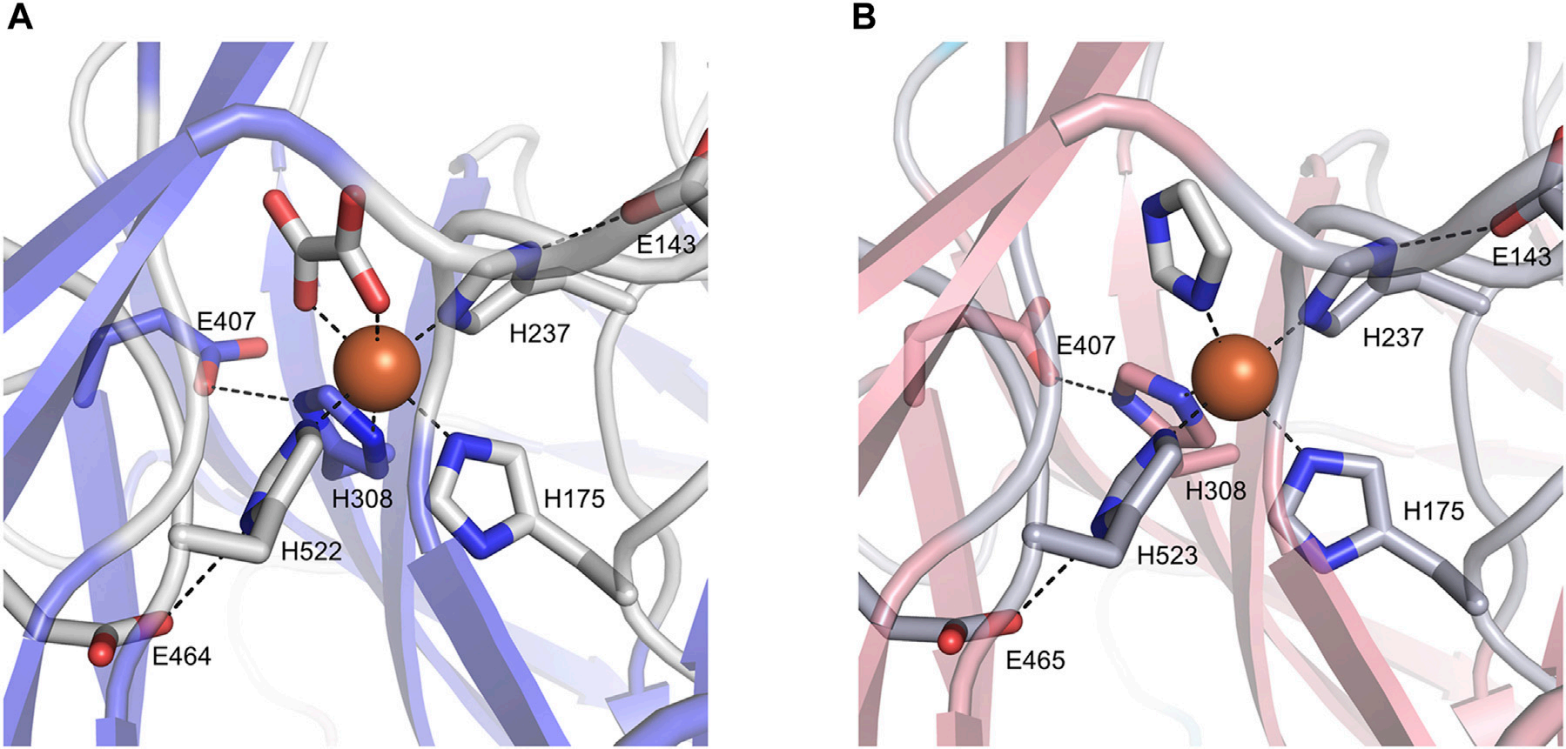
The active centers. **(A)** BCMO-1 active center. Ribbons are in transparence; ligands and the side chains of residues constituting the iron center are shown as sticks; dashed lines denote metal coordination and the hydrogen bonds of Glu143, Glu407, and Glu464/465 with His237, His308 and His522/523, respectively. **(B)** BCMO-2 active center.

The ligand observed in the active center of BCMO-1 was modeled as an oxalate. The dicarboxyl groups and imidazoles from His175, His237, His308, and His522 octahedrally coordinate the iron (Figure 6A). Three second-shell glutamates (Glu143, Glu407, and Glu464) stabilize His237, His308, and His522 via hydrogen bonds. The ligand bound to BCMO-2 was modeled as an imidazole, and the configuration of the BCMO-2 iron center was highly similar to that of BCMO-1 (Figure 6B). It should also be noted that the possibility of small ligand other than oxalate or imidazole cannot be excluded, nor the possibility that the ligands are responsible for the lack of detected activity.

### 3.4 β-Carotene and Apo-β-Carotenal Cleavage Assay

**FIGURE 7:**
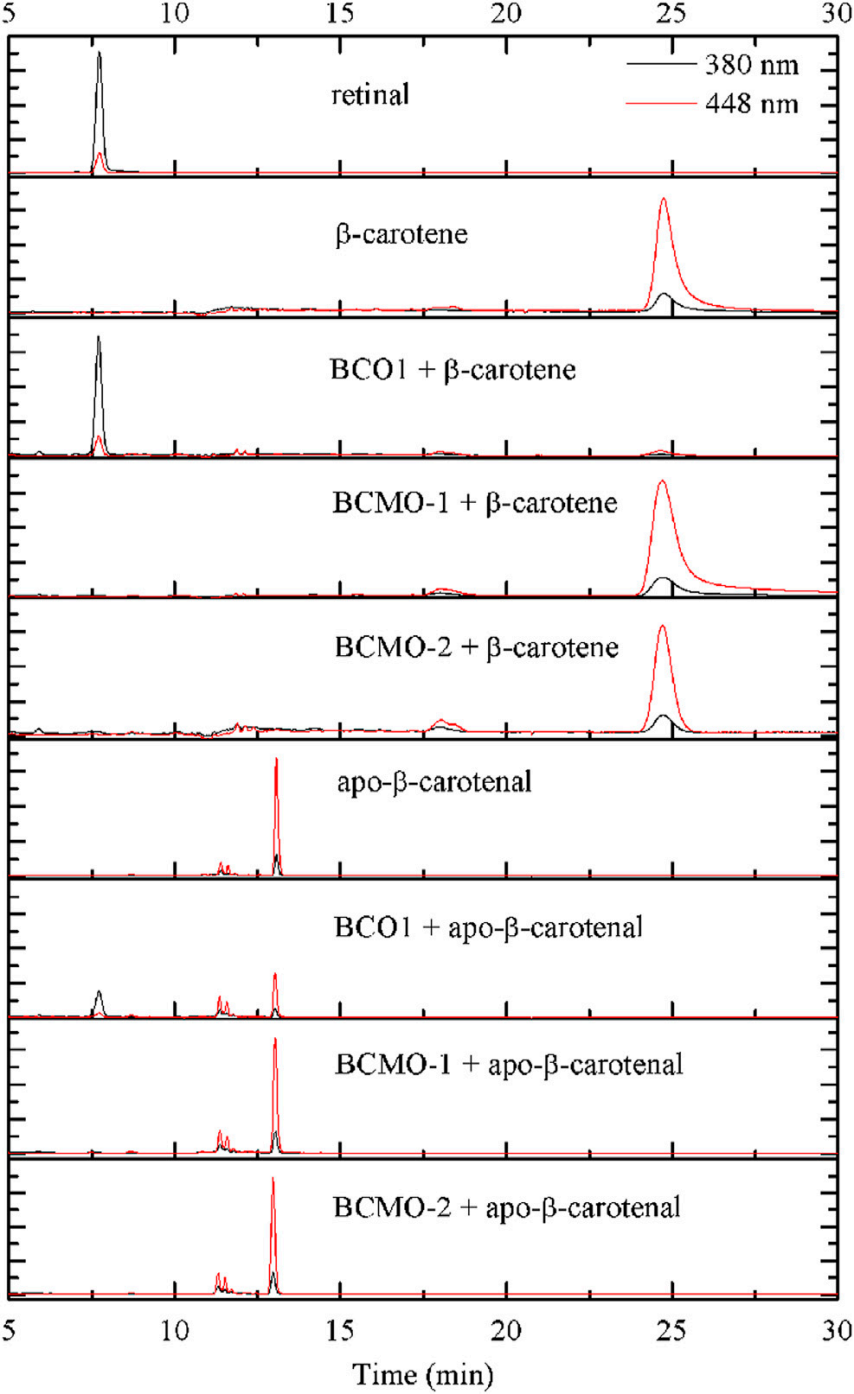
BCMO-1 and BCMO-2 do not cleave β-carotene or apo-β-carotenal. Chromatograms were recorded at 380 nm (black profile) and 448 nm (red profile). Separation of the substrate and product was achieved with propan-2-ol/acetonitrile/water (72/18/10, v/v/v) from time 5–30 min of HPLC analysis (described under Materials and Methods).

The activity of BCMO-1 and BCMO-2 was assayed with β-carotene and apo-β-carotenal using a 12-h reaction time (Figure 7). Mouse BCO1 was used as positive control. Despite the annotation that BCMO-1 and BCMO-2 were β-carotene oxygenases, neither enzymes demonstrated β-carotene cleavage ability. When the asymmetric substrate apo-β-carotenal was tested, no cleaved product was detected either. These results were in contrast to those of mouse BCO1, which cleaved both substrates and produced retinal. The lack of evidence that BCMO-1 and BCMO-2 are able to cleave β-carotene or apo-β-carotenal suggests that these two proteins are not functional orthologs of mammalian BCOs. It should be noted that the current assay conditions are suboptimal and the substrate scope still needs to be addressed.

## 4 Discussion

**FIGURE 8:**
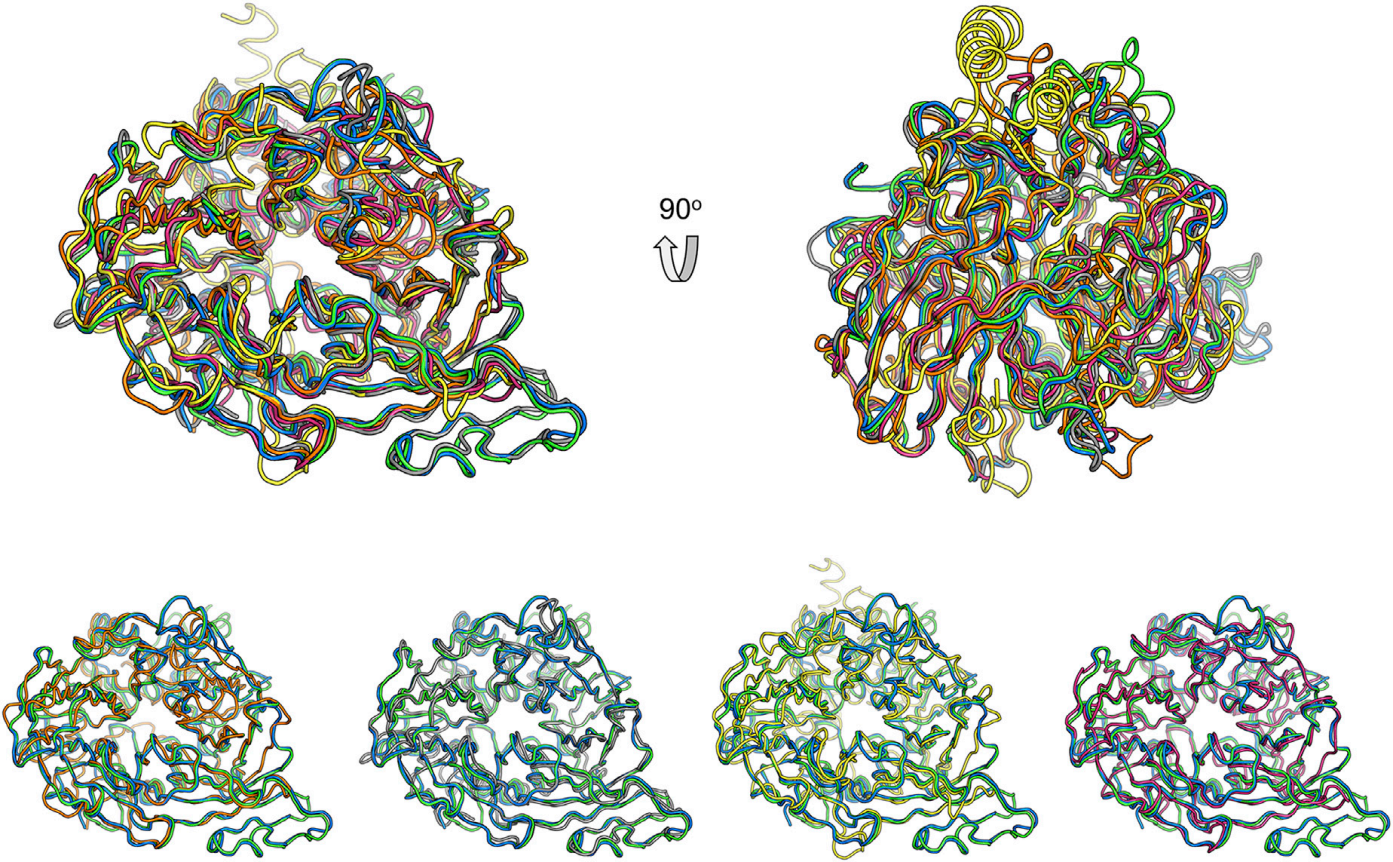
Comparison of BCMO-1 and BCMO-2 with ACO, RPE65, VP14, and NdCCD. Superimposed structures are in tube representation. BCMO-1 and BCMO-2 are in green and blue, respectively. The coordinates of ACO (orange), RPE65 (gray), VP14 (yellow), and NdCCD (purple red) are from PDB entries 2BIW, 4RSE, 3NPE, and 6VCF, respectively. Superimposition of BCMO-1 and BCMO-2 with individual CCD protein is shown in the bottom panel.

Structural comparison with other CCD family members might provide clues for the function of these two nematode proteins. Superimposition of ACO (Kloer et al., 2005), RPE65 (Kiser et al., 2009), VP14 (Messing et al., 2010), and NdCCD (Daruwalla et al., 2020) over BCMO-1 shows that their overall structures are similar (Figure 8). The RMSD values for the aligned Cα atoms between individual superimposed proteins (ACO, RPE65, VP14, and NdCCD) and BCMO-1/BCMO-2 are 1.56/1.71 Å, 1.06/1.08 Å, 2.61/2.97 Å, and 1.15/1.17 Å, respectively, indicating that BCMO-1 and BCMO-2 are more structurally similar to RPE65 and NdCCD than to ACO and VP14. The RMSD values are consistent with the amino-acid sequence identities between individual proteins and BCMO-1/BCMO-2, which are 25%/26%, 34%/33%, 21%/20%, and 31%/30%, respectively. The major variations are located in the helical dome. In addition, BCMO-1, BCMO-2 and RPE65 share an insertion within blade III (including η11 and β27 in BCMO-1 and BCMO-2), which is absent in ACO, VP14, and NdCCD. While the function of the two nematode CCDs still await further characterization, the structures reported here provide the first structural details for animal CCD members.

The physiological function of BCMO-1 has been characterized in *C. elegans* (Cui et al., 2007; Cui and Freedman, 2009), but the knowledge about BCMO-2 is scarce. The strikingly similarity between BCMO-1 and BCMO-2 (with an overall RMSD of 0.39 Å for 429 Cα atoms) highly suggests that they are functional homologs, as also reflected by their high sequence identity of 71%. Such similarities suggest that they originate from a gene duplication event of the nematode line and are phylogenetically diverged from typical CCDs. BCMO-1 is involved in retinoic acid signaling and is expressed in *C. elegans* intestinal cells. Interestingly, intestinal cells are where retinal is detected (Wang et al., 2014). Considering the similarity to RPE65 and our unsuccessful attempt to detect their β-carotene cleavage ability, we propose that the two CCD members may work on some retinoid molecule. Although currently no difference is observed when retinal is tested, this possibility awaits to be addressed in future studies.

In summary, we have solved the structures of BCMO-1 and BCMO-2 that belong to the CCD family. A structural comparison with the known structures of CCDs suggests a role other than β-carotene cleavage, which is consistent with *in vitro* functional data. These two worm CCD members are likely to be functionally similar to the retinoid isomerase.

## Data Availability

The datasets presented in this study can be found in online repositories. The names of the repository/repositories and accession number(s) can be found below: https://www.wwpdb.org/pdb?id=pdb_00007wh0;https://www.wwpdb.org/pdb?id=pdb_00007wh1.
